# How does the tubulin code facilitate directed cell migration?

**DOI:** 10.1042/BST20240841

**Published:** 2025-02-21

**Authors:** Miguel Marques Simoes-da-Silva, Marin Barisic

**Affiliations:** 1 Cell Division and Cytoskeleton, Danish Cancer Institute, Copenhagen, Denmark; 2 Department of Cellular and Molecular Medicine, Faculty of Health and Medical Sciences, University of Copenhagen, Copenhagen, Denmark

**Keywords:** Cell migration, Microtubules, Tubulin code, Tubulin PTMs, Tubulin detyrosination, Tubulin acetylation

## Abstract

Besides being a component of the cytoskeleton that provides structural integrity to the cell, microtubules also serve as tracks for intracellular transport. As the building units of the mitotic spindle, microtubules distribute chromosomes during cell division. By distributing organelles, vesicles, and proteins, they play a pivotal role in diverse cellular processes, including cell migration, during which they reorganize to facilitate cell polarization. Structurally, microtubules are built up of α/β-tubulin dimers, which consist of various tubulin isotypes that undergo multiple post-translational modifications (PTMs). These PTMs allow microtubules to differentiate into functional subsets, influencing the associated processes. This text explores the current understanding of the roles of tubulin PTMs in cell migration, particularly detyrosination and acetylation, and their implications in human diseases.

## Dynamic instability of microtubules

Microtubules are tubular structures that serve as a major component of eukaryotic cytoskeleton. They are formed as polymers of α- and β-tubulin heterodimers in a head-to-tail arrangement, which provides their intrinsic polarity. Tubulin heterodimers are stacked into 13 protofilaments, which together form the 25-nm-diameter hollow tubes. This conformation grants high mechanical resistance, allowing the formation of larger structures [1], such as the mitotic/meiotic spindle and the axonemes that are essential for correct cell division and cilium and flagellum movement, respectively [2–7]. In neurons, microtubules also play a vital role in establishing and maintaining the complex network of neuronal connections, facilitating transport along axons and dendrites [8–10].

Despite their well-defined fundamental structure, microtubules are some of the most dynamic components of the cells. This dynamicity largely relies on a property known as the dynamic instability [11], characterized by the switching of growth to shrinkage (catastrophe) and shrinkage to growth (rescue) states of microtubules. The rescue depends on addition of GTP-tubulin subunits to the growing microtubule end and its subsequent hydrolysis to GDP-tubulin within the microtubule lattice. The incorporation of GTP-tubulin subunits creates a protective ‘GTP cap’, the loss of which leads to microtubule catastrophe.

However, microtubule dynamics and variability go far beyond the process of their formation, greatly depending on genetic and chemical factors, which constitute a group of variations widely designated as the ‘tubulin code’.

## The tubulin code

The concept of the ‘tubulin code’ traces its roots to the ‘multi-tubulin hypothesis’ proposed by Fulton and Simpson in 1976. Their hypothesis was based on observations made while studying the amoeboflagellate *Naegleria*, in which they discovered that tubulin proteins exhibit different antigenic properties in various stages of the organism’s life cycle. They proposed that an organism’s genome encodes multiple α- and β-tubulin isoforms, each with distinct, nonredundant functions, which could be selectively expressed by the cell [12,13]. With the development of genome analysis techniques such as comparative sequence analysis [14] and, in recent years, whole-genome sequencing, this was proven to be correct. Many organisms have multiple genes encoding distinct isoforms of α- and β-tubulin, with the human genome containing nine isoforms of each α- and β-tubulin [15–17].

While tubulin isoforms significantly contribute to the functional diversity of microtubules, they represent only part of the complexity underlying microtubule behavior. Since the early 21st century, the concept of the ‘tubulin code’ has emerged, incorporating the additional variability introduced by tubulin post-translational modifications (PTMs) [15,18].

## Tubulin post-translational modifications

Although many tubulin PTMs were identified half a century ago [19–21], it was the discovery and characterization of the enzymes responsible for these modifications in the following decades that truly advanced our understanding of how these PTMs regulate microtubule dynamics and functionality [15–17].

**Figure 1:**
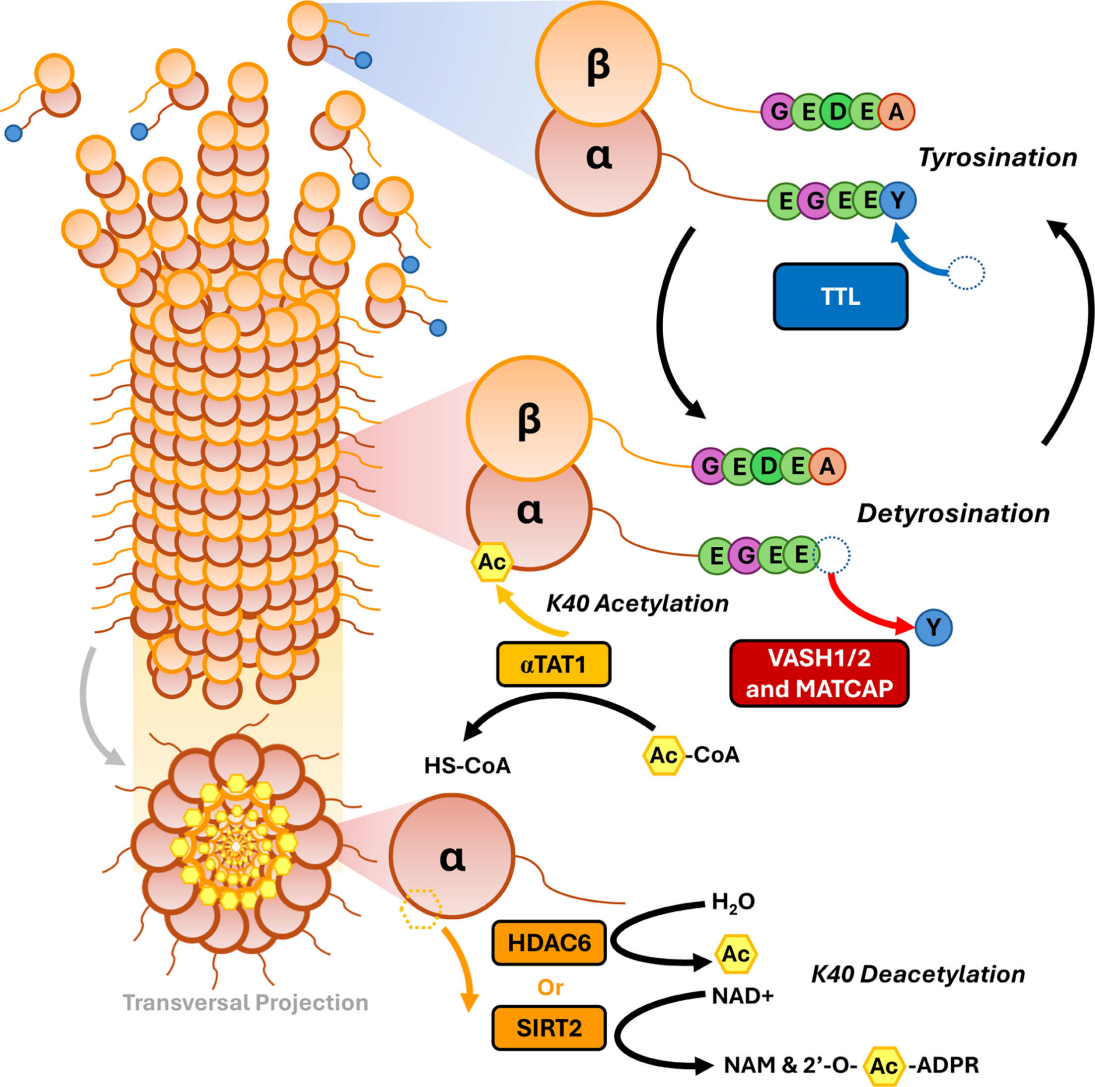
Tubulin PTMs extensively studied in cell migration. Representation of the processes of acetylation and detyrosination/tyrosination cycle. αTAT1 can use an acetyl group from acetyl-CoA to add it to the K40 lysine of α-tubulin in the lumen of the microtubule, while HDAC6 and SIRT2 perform the reverse, deacetylation process. Detyrosination occurs at the outer surface of the microtubule, where the C-terminal tyrosine (Y) of α-tubulin is removed by VASH1, VASH2, or MATCAP. This process is then reversible, with TTL adding back the tyrosine residue to soluble tubulin dimers. Abbreviations: αTAT1, α-tubulin acetyltransferase 1; HDAC6, histone deacetylase 6; MATCAP, microtubule-associated tyrosine carboxypeptidase; PTMs, post-translational modifications; SIRT2, sirtuin 2; TTL, tubulin tyrosine ligase; VASH, vasohibins.

Several tubulin PTMs, such as phosphorylation, methylation, and ubiquitylation [19,22,23], were already known to occur in other proteins. However, some modifications, like the removal or addition of residues at the C-terminal end, along with (poly)glycylation and (poly)glutamylation, were first identified in tubulin, with the latter two later found to modify other substrates as well [20,21,24–28]. While some tubulin PTMs, such as acetylation and phosphorylation, occur at the globular ‘body’ of tubulin, most of them affect the external C-terminal tails of tubulin, which radially project outward from the cylindrical lattice of the microtubule [16] (Figure 1).

Tubulin PTMs can be read by a variety of motor proteins and microtubule-associated proteins (MAPs), whose distribution and activity are consequently modulated. For instance, kinesin-1 and kinesin-2 displayed increased processivity and velocity along highly glutamylated microtubules [29]. Glutamylation also enhances the activity of microtubule-severing proteins such as spastin [30,31] and katanin [32], as well as the microtubule polymerase Elongator [33]. In contrast, glycylation antagonizes glutamylation by inhibiting katanin activity [32]. Microtubule plus-end-tracking proteins such as CLIP-170 and the dynactin subunit p150(Glued) are specifically recruited to tyrosinated tubulin through their CAP-Gly domains [34], which is a critical step for initiating dynein-driven transport [35,36]. Additionally, the MAP EML2-S binds to tyrosinated tubulin to regulate microtubule dynamics at shrinking plus-ends [37]. The following chapters will delve deeper into the readers of acetylation and detyrosination.

Beyond acting as readers of the tubulin code, MAPs also regulate tubulin PTMs, further adding to the complexity of interactions between tubulin isoforms, PTMs and MAPs. For instance, MAP7 was shown to promote tubulin acetylation while simultaneously preventing detyrosination [38], whereas Calmodulin-regulated spectrin-associated proteins CAMSAP2 and CAMSAP3 were shown to promote detyrosination [39].

This article explores the role of the tubulin code in cell migration. While other tubulin PTMs, such as glutamylation [40] and tri-methylation [41], along with certain tubulin isoforms [42], were shown to affect migration-associated processes, we focus on two PTMs with the most compelling evidence supporting their involvement in cell migration: acetylation and detyrosination. The substantial body of evidence associated with these two PTMs can largely be attributed to the availability of specific tools and antibodies that have enabled extensive research on these modifications.

## Tubulin acetylation

The acetylation of the lysine 40 (K40) residue of α-tubulin was originally identified by L’Hernault and Rosenbaum in 1985 [43]. However, it was only in 2010 that researchers identified the enzyme responsible for the K40 acetylation, the α-tubulin acetyltransferase 1 (αTAT1) [44,45]. The modification was shown to occur exclusively in polymerized tubulin [46–49], leading to questions about how acetylation could occur in the microtubule lumen [50]. Current evidence suggests that αTAT1 accesses the microtubule lumen both through its ends [51,52] and via defects in the protofilament lattice [51,53]. Once inside, αTAT1 promotes increased microtubule acetylation. Conversely, deacetylation of tubulin can be performed by two enzymes: histone deacetylase 6 (HDAC6) and sirtuin 2 (SIRT2), each of which targets both soluble tubulin dimers and microtubules [54–56] (Figure 1).

Although α-tubulin K40 acetylation is known to be associated with a subset of more stable microtubules, its effect on the mechanical properties of microtubules still remains unclear. Recent structural studies revealed that the acetylation of the luminal unstructured loop containing K40 reduces interprotofilament interactions, thereby enhancing microtubule flexibility [57]. This increased flexibility protects microtubules from acquiring lattice damage due to repeated bending (also known as mechanical aging); thus, it reduces breakage and extends their lifespan [58,59]. For instance, acetylation increases in maturing axonemes of cilia and flagella, possibly protecting against movement-induced aging [45,60–62].

Acetylation regulates various cellular processes, including microtubule-based cargo transport, particularly in neurons [63–66]. However, studies using αTAT1-knockout mice and acetylation-blocking drugs have shown only mild effects on these processes, such as reduced touch sensation [67–70]—a finding consistent with previous studies in *Drosophila melanogaster* and *Caenorhabditis elegans* [44,71–73].

## Tubulin detyrosination and retyrosination

The discovery of α-tubulin tyrosination in the 1970s marked the first known instance of an RNA- and ribosomes-independent amino acid addition to a protein [20,74]. Soon after, it was revealed that the primary modification was detyrosination—the removal of the genetically encoded C-terminal tyrosine from α-tubulin—and that the initially observed phenomenon was actually retyrosination, part of a reversible cycle [24,75]. Still, it is of note that α-tubulin isoform TubA4a does not intrinsically encode a C-terminal tyrosine, and thus the primary modification in this isoform would be tyrosination.

Shortly after tyrosination was described, Raybin and Flavin identified the enzyme responsible for this process, the tubulin tyrosine ligase (TTL), which reattaches tyrosine to the C-terminal glutamate of α-tubulin [76]. Nevertheless, it took another 40 years to identify the enzymes responsible for detyrosination, historically known as tubulin carboxypeptidases. In 2017, vasohibins VASH1 and VASH2, along with small vasohibin-binding protein (SVBP), were found to detyrosinate α-tubulin [77,78]. More recently, a third enzyme, named microtubule-associated tyrosine carboxypeptidase, was also discovered to cause detyrosination [79] (Figure 1).

Research on the target specificity of these enzymes revealed that TTL selectively retyrosinates soluble tubulin heterodimers [76,80], whereas VASH1/2 target polymerized tubulin within the microtubules [77,81]. Due to this specificity, longer lived microtubules become increasingly detyrosinated, while TTL rapidly retyrosinates depolymerized tubulin dimers [80] (Figure 1).

The detyrosination/retyrosination cycle plays diverse cellular roles. Similar to acetylation, some studies suggest a positive role in regulating microtubule flexibility, as observed in heart and skeletal muscle contraction [82,83]. However, it still remains unclear whether this effect is direct or involves other components.

In neurons, the detyrosination/tyrosination balance is crucial for neurodevelopment and neuronal connectivity, as evidenced by the perinatal death of TTL-knockout mice due to neurodevelopmental defects [84]. Moreover, mutations in VASH1/2 cofactors, associated with conditions like microcephaly and intellectual disability, further highlight the importance of this balance [85,86].

Another important role of the detyrosination/tyrosination cycle was revealed in skeletal and heart muscle microtubules, where it affects mechanotransduction [82]. In heart muscle fibers, where microtubules buckle with each heartbeat, detyrosination enhances muscle viscoelasticity by linking the microtubules to desmin [83]. Accordingly, lack of detyrosination impaired cardiac muscle function, while excessive detyrosination has been associated with stiffer cardiac muscles and heart failure [87].

During cell division, detyrosination is enriched in the microtubules of the mitotic spindle oriented toward the spindle equator [88], where it promotes kinesin-7/CENP-E motor activity, facilitating the transport of unaligned chromosomes to the metaphase plate [89,90]. The detyrosination/tyrosination balance was also shown to be critical for regulating the mitotic functions of the microtubule depolymerase Mitotic centromere-associated kinesin (MCAK), a member of the kinesin-13 proten family. MCAK activity at centromeres facilitates the release and remodeling of microtubule attachments, which is crucial for correcting spindle attachment errors during cell division [91]. By negatively regulating MCAK-mediated depolymerization [92], excessive detyrosination prevented the correction of erroneous kinetochore–microtubule attachments [93]. Moreover, increased detyrosination was proposed to disrupt MCAK’s role in maintaining proper spindle orientation during mitosis [34,94].

A recent study revealed another mitotic role of detyrosination/tyrosination cycle. By modulating Cytoplasmic linker associated protein 2 (CLASP2) and NDC80 binding to spindle microtubules, the levels of detyrosinated/tyrosinated α-tubulin near kinetochore microtubule plus-ends regulate chromosome oscillations associated with timely anaphase onset [95].

## The microtubule cytoskeleton and its role in cell migration

The cytoskeleton is a complex intracellular network, mainly composed of actin filaments, intermediate filaments, and microtubules. The profound cross-talk between these cytoskeletal components plays a crucial role in diverse cellular processes, including cell migration [96]. The association between the microtubule cytoskeleton and eukaryotic cell movement was first reported in 1970 [97], when it was demonstrated that microtubule-depolymerizing agents hindered the formation of the cellular leading edge and impaired directed movement in fibroblasts. During the following decades, many studies have expanded the understanding of microtubules’ effect on motility, with focus on their roles in intracellular trafficking and organizing structural and signaling compounds [98–100].

Cell migration studies using 2D cell cultures have played a fundamental role in understanding how microtubules affect the formation and degradation of focal adhesions and, together with actomyosin activity, regulate directed movement [101]. However, movement in the 3D environments, which is found all throughout the body of a multicellular organism, while sharing similarities with the former, also possesses its own additional complexities. These cells are surrounded by an extracellular matrix (ECM), which can vary widely in properties and composition. During migration, cells must adjust adhesion dynamics to properly accommodate the differences in surrounding environment [101]. Another way to study cell migration is via the so-called 1D migration assays, where cell movement is confined to a single direction using micropatterned lines. Compared with 2D assays, 1D migration assays not only simplify data analysis but also more closely resemble the physiological conditions associated with 3D migration assays [102].

Cell migration has been generally divided into two main types: (1) amoeboid migration, which is prominent in immune cells, requiring fewer adhesions and characterized by bleb-like protrusions driven by actomyosin contractility [103,104]; and (2) mesenchymal migration, which involves stronger cell–cell and cell–ECM interactions, with cytoskeletal components, including microtubules, polarized in the direction of movement, where strong ECM-degrading protease activity is present [105,106]. However, recent studies challenge this binary classification, revealing migration plasticity that allows for hybrid strategies, such as the nucleus movement-dependent lobopodial migration, which are crucial for responding to complex ECM dynamics [96,107–111]. While the microtubule cytoskeleton plays a vital role in both migration types, current research has been focused primarily on its effect in mesenchymal migration [112,113]. Here, we will discuss the microtubule cytoskeleton’s role in both 2D and 3D environments, with an emphasis on mesenchymal migration.

## Cell polarization and microtubule-based directed transport

**Figure 2:**
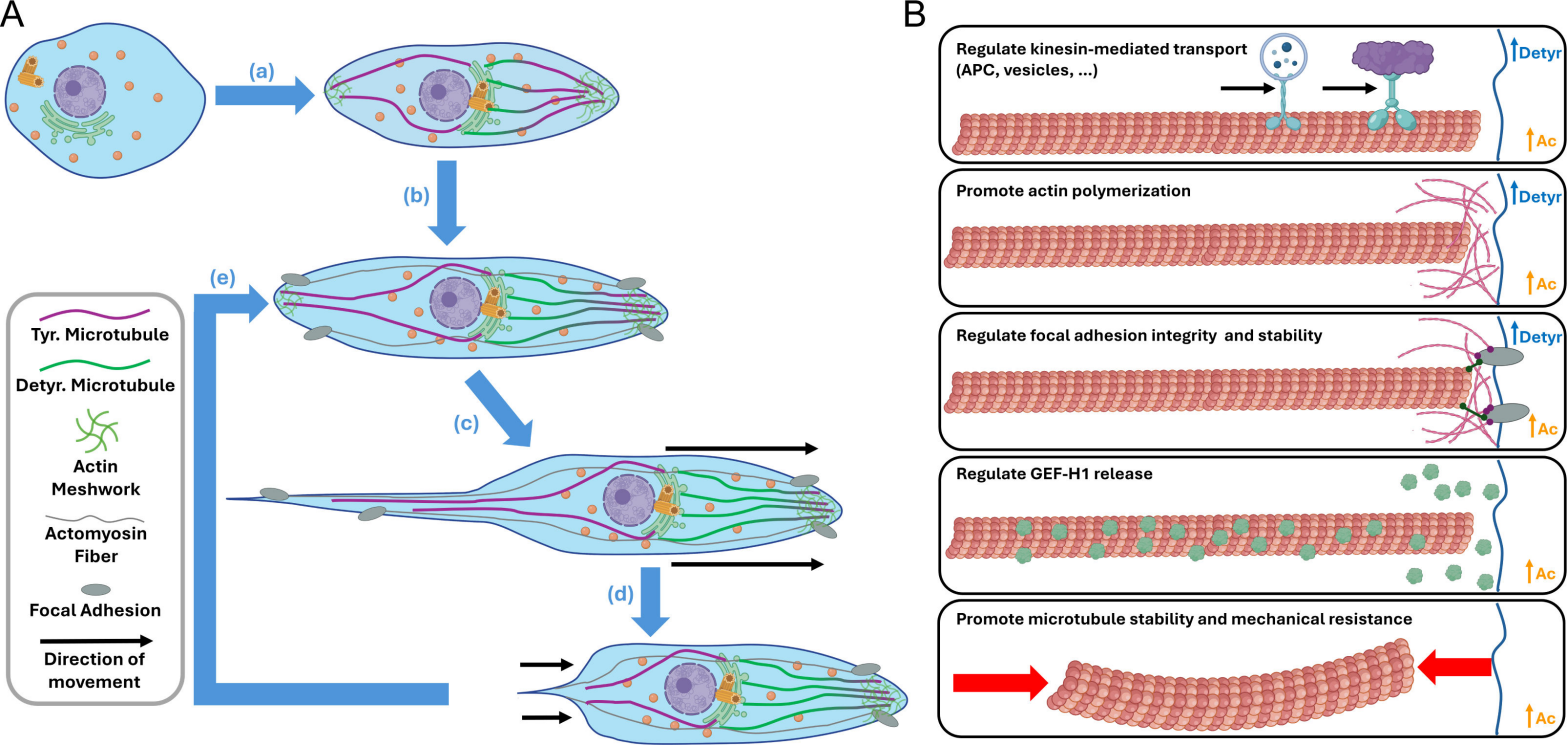
The roles of tubulin PTMs in cell migration. (**A**) Representation of the steps of directed mesenchymal cell migration: (a) The cell polarizes, moving the centrosome and the Golgi complex to one side of the perinuclear region, and rearranging its microtubule and actin cytoskeleton, forming a defined front and rear. (b) The cell then stretches out with the help of microtubule and actin polymerization and attaches to the extracellular substrates through focal adhesions. (c) Through forward-directed forces transmitted via the focal adhesions by actomyosin contractility, the cell front is pushed forward. (d) The focal adhesions at the cell rear are dissolved, allowing the cell rear to be retracted by further actomyosin contractility. (e) This process then repeats cyclically, allowing for continued movement in a defined direction. Note that detyrosinated tubulin (depicted in green) decorates longer living microtubules that point toward the cell leading edge. (**B**) Detyrosination and acetylation play crucial roles during cell migration. Detyrosinated microtubules promote kinesin-1 transport of essential polarization markers, such as APC, while tubulin acetylation has also been reported to play a role in regulating kinesin movement. Both modifications have also been shown to regulate the polymerization of actin fibers at the cell edges, as well as focal adhesion formation and stability. Acetylation also regulates GEF-H1 release (important for Rho GTPase signaling) and provides mechanical resistance and structural stability to the microtubules. Abbreviations: APC, adenomatous polyposis coli (APC); PTMs, post-translational modifications (PTMs).

Symmetry breaking and cell polarization are required to establish a front leading edge and a rear trailing edge during directed cell migration. To achieve this and maintain the mesenchymal migration, cytoskeleton components, including microtubules, must organize at the leading edge [105,106]. Microtubule polymerization is already intrinsically polar, with a fast-growing plus-end, where the β-tubulin is exposed, and a more static minus-end, where α-tubulin is exposed [114]. During mesenchymal migration, plus-ends orient toward cell extremities, while microtubule organizing centers, such as the centrosome and Golgi apparatus, position themselves in front of the nucleus, directing microtubule polymerization toward the leading edge [115,116] (Figure 2A).

This polarized microtubule organization is essential for directed transport of effector and signaling molecules, mRNAs, and vesicles to the leading edge; most of which are transported by plus-end-directed motor proteins, kinesins [117], and further serve as polarizing factors, maintaining cellular asymmetry. The leading edge-specific mRNAs primarily code for actin regulators like the Arp2/3 complex [118,119], while secretory vesicles provide integrins, cytoskeletal components, and lipid membranes necessary for cell elongation and protrusion formation [120–124]. Simultaneously, microtubules support the formation of protrusions by providing pushing forces and resistance to compression, which further facilitates forward cell movement [125,126] (Figure 2B).

## Microtubule–actin cross-talk: regulation of adhesion dynamics and generation of migration forces

One of the crucial factors in regulating cell migration is the cross-regulation between microtubules and actin filaments/actomyosin complexes. For instance, microtubule ends directly facilitate actin nucleation and polymerization via a microtubule plus-end-tracking protein (+TIP) adenomatous polyposis coli (APC) [127,128]. APC is transported by kinesin-1 and kinesin-2 to the microtubule plus-ends [129], where it binds to +TIP complexes via EB1 [130,131]. This facilitates APC accumulation at the cell’s leading edge, where it both stabilizes microtubules and promotes actin nucleation and polymerization [132], highlighting the complex interplay between these cytoskeletal components.

Another common element that co-ordinates microtubule and actin dynamics during migration is the Rho family of GTPases [133]. When bound to microtubules, Rho guanine nucleotide exchange factor (Rho GEF) proteins remain inactive [134,135]. However, dynamic instability at microtubule ends allows Rho GEFs to be released, activating RhoA into its GTP-bound form. Active RhoA then promotes actomyosin contractility required for cell movement, while also stabilizing the focal adhesions connected to the actin fibers [135–137]. In contrast, microtubule polymerization and stability stimulate Rac1 activity, which, in turn, enhances actin filament growth and microtubule assembly, thereby increasing cell protrusions [138–141]. This counterbalance between Rho and Rac GTPases is crucial for regulating the cytoskeleton and enabling proper cell migration.

During directed cell migration, CLASP proteins tether the microtubules near focal adhesion sites [142–144], facilitating the transport of key molecules required for focal adhesion formation and turnover, including integrins [145–150]. Moreover, microtubules regulate focal adhesions by modulating the transport and activity of APC, RhoA, and Rac1 [135–137]. By sequestering the Rho GEF protein GEF-H1 [151] and transporting relaxation factors [152,153], microtubules also facilitate focal adhesion disassembly at the cell’s trailing edge (Figure 2B).

The process of cell migration can be summarized in the following key steps: (1) cell polarization, (2) protrusion of the leading edge, (3) formation of new focal adhesions at the cell front, (4) actomyosin-driven forward movement of the cell body, coupled to (5) retraction of the rear (Figure 2A). As outlined above, the microtubule cytoskeleton directly or indirectly regulates all these steps. The precise and dynamic control of directed cell migration by microtubules is largely attributed to the plasticity provided by PTMs, which we discuss next.

## The impact of tubulin acetylation on cell migration

Acetylation of α-tubulin at K40 plays a critical role in enhancing microtubule flexibility and stability, which, in turn, influences cell migration in various ways. A recent study found that acetylation, along with CLASP proteins, accumulates in damaged microtubule regions in response to tensile and contractile forces encountered during migration. This accumulation plays a critical role in repairing and reinforcing microtubules, which is essential for enabling the cell to deform its structures, such as the nucleus, in order to successfully migrate through confined and constricted spaces [154] (Figure 2B).

Another key function of α-tubulin acetylation is the regulation of Rho GEF-H1 release, essential for controlling actin polymerization and focal adhesion dynamics. If this release were solely dependent on microtubule depolymerization, it would trigger widespread GEF-H1 activation that disrupts directed cell migration, as observed in nocodazole-treated cells with fully depolymerized microtubules [155]. However, recent studies suggest that localized microtubule acetylation fine-tunes spatial GEF-H1 release, facilitating precise microtubule–actin cross-talk during migration [156,157]. Stabilized, acetylated microtubule regions appear to increase GEF-H1 activation compared with deacetylated regions, indicating that GEF-H1 release depends not only on microtubule depolymerization but also on mechanical changes in response to external cues (Figure 2B).

By promoting Rho GEF-H1 release and activation, microtubule acetylation enhances cell contractility and migration forces at focal adhesions, particularly in response to physically demanding substrates. For instance, the acetylating enzyme αTAT1 interacts with a mechanosensitive component of focal adhesions, talin, influencing mechanotransduction at focal adhesions [156] (Figure 2B). Relying on GEF-H1-dependent contractile forces and αTAT1 activity, KN motif- and ankyrin repeat domain-containing protein 1 (KANK1) mediates the connection between microtubules and talin, promoting adhesion sliding and disassembly during cell polarization and migration [158]. Notably, αTAT1-mediated acetylation at adhesion sites influences the formation, turnover, and spatial organization of focal adhesions, as well as the transport of vesicles carrying focal adhesion components, a process critical for effective cell migration in astrocytes [159]. Furthermore, the acetylation-induced increase in microtubule flexibility and mechanical resilience enables migrating cells to penetrate confined spaces by adapting to ECM [160].

Acetylation has also been proposed to regulate kinesin-mediated intracellular transport that may play important roles during cell migration (Figure 2B). Several studies suggest that acetylation enhances kinesin-1-mediated transport [63,161–165], while diminishing the transport by others, such as kinesin-3 [166]. However, the mechanism by which this intraluminal modification affects the microtubule lattice where MAPs and motors bind still remains unclear. Given the importance of precise transport to the cell’s leading and trailing edges in maintaining cell polarization during directed migration, a differential balance of microtubule acetylation in these regions could significantly affect both polarization and migration efficiency.

Acetylation was also associated with cancer cell invasion capacity. A study showed that αTAT1 localizes to the invadopodia of breast cancer cells and is crucial both for their 2D migration and invasive migration through a collagen matrix. αTAT1 also facilitates the transport of vesicles containing MT1-MMP metalloproteinases, enzymes essential for matrix degradation during cancer cell invasion [167]. Given the importance of these processes in cancer cell migration and metastasis, a deeper understanding of the acetylation/deacetylation balance could provide valuable insights for developing targeted therapeutic strategies.

## The impact of tubulin detyrosination on cell migration

Accurate distribution of specific factors to the leading edge of a migrating cell largely depends on microtubule plus-end-directed transport by kinesins. However, due to the microtubule orientation within the cell [114], kinesins can transport cargo to plus-ends pointing toward both leading and trailing edges.

This raises a critical question: how does the cell precisely direct cargo toward the leading edge in order to establish and maintain the polarity needed for directed migration? Emerging evidence suggests that tubulin PTMs play a pivotal role in this process. In addition to the previously discussed tubulin acetylation, the detyrosination/tyrosination balance appears to be critical for guiding kinesin-driven transport during directed cell migration (Figure 2B). Multiple kinesin motors, including kinesin-1 and kinesin-2, show a preference for stable, highly detyrosinated microtubules [29,164]. In contrast with the intraluminal location of acetylation, detyrosination occurs along the outer microtubule lattice, at the α-tubulin C-terminal tails, which directly interact with motor proteins and MAPs (Figure 1).

Pioneering research by Gundersen and Bulinski showed that microtubules in the front of migrating cells are more detyrosinated compared with those at the rear [168] (Figure 2A). A recent study has further explored the role of microtubule detyrosination in processes critical for leading edge formation. Detyrosination was found to spatially direct kinesin-1-mediated transport of APC, causing its accumulation at the leading edge, where it promoted actin polymerization and microtubule stability, thus facilitating symmetry breaking for directed cell migration [169]. This detyrosination-dependent regulation of actin and microtubule dynamics further affects the formation of focal adhesions, which are critical for cell motility (Figure 2B).

APC transport plays a vital role in cell migration in both healthy and cancer cells. In colorectal cancer cells, APC regulates actin filament nucleation essential for cell junction dynamics and integrity [170]. Disruption of APC impairs these processes, leading to defective cellular shape and loss of directional migration. Moreover, APC-mediated actin nucleation, along with formins and the Arp2/3 complex, is involved in forming invasive protrusions in tumor cells, a critical step in metastasis initiation [171]. In immune cells like T lymphocytes, APC disruption hinders directed migration by affecting cytoskeletal organization, adhesion formation, and polarization, all of which are vital for an effective immune response [172].

APC also facilitates peripheral localization of specific RNAs, which are transported to cell protrusions via kinesin-1 along detyrosinated microtubules [98,173,174]. These APC-dependent RNAs influence cell migration [98,175], and their localization to protrusions is enhanced by substrate stiffness and cellular mechanoactivity [98,176]. Such RNAs, like *RAB13*, play an active role in promoting cell type-dependent migration within confined environments [98,175,176].

The equilibrium between detyrosinated and tyrosinated microtubules plays an important role in apicobasal polarization and cell migration during epithelial monolayer formation and ciliogenesis [177,179]. This equilibrium regulates migration direction, cell morphology, cilia length, and adhesion formation. In addition, increased microtubule detyrosination facilitates transport of cellular components to the apical membrane [177], further demonstrating how this PTM establishes cell polarity and directs microtubule-based transport in polarized cells.

The dysregulation of the microtubule detyrosination/tyrosination balance has been implicated in various diseases where cell polarity and migration are critical. Loss of this balance can impair neuron migration and axonal growth, which may be linked to defects in VASH1/2-SVBP observed in certain neurodevelopmental conditions [77,84,86,180].

In cancer, dysregulated detyrosination has been associated with angiogenesis [181] and the ability of the cells to undergo epithelial-to-mesenchymal transition, a crucial step for cancer cell invasiveness and metastasis formation [182,183]. Abnormal detyrosination can enhance these processes, thereby promoting tumor progression.

Additionally, recent research has linked kinesin-1-mediated transport of mRNAs and ribosomes along detyrosinated microtubules to hypertrophic cardiomyopathy [184].

These associations underscore the importance of further research on the role of detyrosination in cell migration and polarity, which could improve strategies for combating these conditions.

## Conclusions and future perspectives

Even though the role of tubulin PTMs in regulating cytoskeleton and cell migration has been debated for decades, it is only with recent advancements in enzyme identification, microscopy, and structural analysis techniques that we have begun to understand this complex relationship in more detail.

To advance this knowledge, future studies should focus on identifying the missing MAPs that detect and respond to tubulin PTMs. While significant progress has been made in identifying the enzymes responsible for various tubulin PTMs, many questions remain regarding the regulation and spatial co-ordination of these enzymes within cells. Uncovering their regulatory mechanisms along with elucidating the complex interplay between tubulin isotypes, PTMs and MAPs are essential for understanding the role of tubulin PTMs in cell migration.

Moreover, while some studies have been conducted in 3D or biomimetic environments, much of the data still comes from simpler 2D cell cultures. Leveraging recent advancements in 3D and *in vivo* labeling, detection, and imaging technologies will be critical for deepening our understanding of the impact of tubulin PTMs on cell migration in more physiologically relevant contexts.

**Perspectives**

- Tubulin post-translational modifications (PTMs) play a critical role in regulating microtubule properties and microtubule-based intracellular transport required for directed cell migration.
- Tubulin PTMs, such as acetylation and detyrosination, play crucial rolls during migration. They do so by enhancing and directing kinesin-1-based intracellular transport of essential polarization markers, by regulating the focal adhesion dynamics and the polymerization of actin fibers at the cell edges, by regulating GEF-H1 release (important for Rho GTPase signaling), and by providing mechanical resistance and structural stability to the microtubules.
- While significant progress has been made in identifying the enzymes responsible for various tubulin PTMs, the motors and MAPs that read and respond to the information written by these enzymes are still largely unidentified. Identifying new readers is essential for understanding the role and underlying mechanisms of tubulin PTMs in cell migration.

## Open Access

This article has been published open access under our Subscribe to Open programme, made possible through the support of our subscribing institutions, learn more here: https://portlandpress.com/pages/open_access_options_and_prices#conditional

## Abbreviations

APC: adenomatous polyposis coli
ECM: extracellular matrix
MAPs: microtubule-associated proteins
PTMs: post-translational modifications
SVBP: small vasohibin-binding protein
TTL: tubulin tyrosine ligase
αTAT1: α-tubulin acetyltransferase 1
